# Estimating Orientation Using Magnetic and Inertial Sensors and Different Sensor Fusion Approaches: Accuracy Assessment in Manual and Locomotion Tasks

**DOI:** 10.3390/s141018625

**Published:** 2014-10-09

**Authors:** Elena Bergamini, Gabriele Ligorio, Aurora Summa, Giuseppe Vannozzi, Aurelio Cappozzo, Angelo Maria Sabatini

**Affiliations:** 1 Interuniversity Centre of Bioengineering of the Human Neuromusculoskeletal System, Department of Movement, Human and Health Sciences, University of Rome “Foro Italico”, P.zza Lauro de Bosis 15, 00135 Roma, Italy; E-Mails: elena.bergamini@uniroma4.it (E.B.); aurora.summa@uniroma4.it (A.S.); aurelio.cappozzo@uniroma4.it (A.C.); 2 The BioRobotics Institute, Scuola Superiore Sant'Anna, Piazza Martiri della Libertà 33, 56124 Pisa, Italy; E-Mails: g.ligorio@sssup.it (G.L.); angelo.sabatini@sssup.it (A.M.S.)

**Keywords:** 3-D orientation, accuracy, wearable sensors, IMU, MIMU, Kalman filtering, gait, upper body, biomechanics, human

## Abstract

Magnetic and inertial measurement units are an emerging technology to obtain 3D orientation of body segments in human movement analysis. In this respect, sensor fusion is used to limit the drift errors resulting from the gyroscope data integration by exploiting accelerometer and magnetic aiding sensors. The present study aims at investigating the effectiveness of sensor fusion methods under different experimental conditions. Manual and locomotion tasks, differing in time duration, measurement volume, presence/absence of static phases, and out-of-plane movements, were performed by six subjects, and recorded by one unit located on the forearm or the lower trunk, respectively. Two sensor fusion methods, representative of the stochastic (Extended Kalman Filter) and complementary (Non-linear observer) filtering, were selected, and their accuracy was assessed in terms of attitude (pitch and roll angles) and heading (yaw angle) errors using stereophotogrammetric data as a reference. The sensor fusion approaches provided significantly more accurate results than gyroscope data integration. Accuracy improved mostly for heading and when the movement exhibited stationary phases, evenly distributed 3D rotations, it occurred in a small volume, and its duration was greater than approximately 20 s. These results were independent from the specific sensor fusion method used. Practice guidelines for improving the outcome accuracy are provided.

## 1. Introduction

The quantitative observation of how humans move provides information concerning both the functions of the locomotor sub-systems and the overall strategy with which a motor activity is executed. An understanding of these functions and strategies can be gained from measurements provided by motion capture techniques, associated with mathematical models of the anatomy and physiology of the organs and systems involved. The validity and reliability of the scientific approach used to achieve this objective, as well as its cost effectiveness, are crucial issues that must be addressed.

In the above-mentioned context, the accurate determination of the three-dimensional (3D) orientation of a body segment, relative to a global Earth-fixed reference frame, is of basic importance. An increasing number of clinicians evaluate the functional outcome of treatments of the locomotor apparatus analyzing joint kinematics and kinetics [1–4]. Body segment orientation is crucial also when monitoring activities of daily living in elderly people for walking instability evaluation and fall risk assessment [5,6]. Again, rehabilitation using virtual/augmented reality requires accurate information about body segment orientation in real time [7].

Several technologies are available for the estimation of the 3D orientation of a rigid body, based on optical, acoustic, mechanical, or magnetic and inertial sensors [8,9]. Among them, magnetic and inertial measurement units (MIMUs) are gaining momentum as they have the advantage of being small, portable, and wireless, thus allowing for unconstrained motion monitoring [10,11]. In addition they are appropriate for real-time applications and are relatively low-cost. Accuracy, however, is still an issue [12].

MIMUs consist of orthogonally mounted single-, two- or three-axis gyroscopes, accelerometers and magnetic sensors, providing the values of angular velocity, the sum of gravitational and inertial linear accelerations, and local magnetic field vector components, about and along the axes of a Cartesian coordinate system fixed with the MIMU (unit local frame: ULF).

The 3D orientation of a MIMU may be estimated by numerical time-integration of the kinematic differential equations that relate the time derivatives of the selected orientation parameters to the angular velocity provided by the gyroscope. The accuracy of this numerical integration is hindered by errors that grow over time due to gyroscope bias drift [13,14]. Moreover, the initial conditions of the integration process are unknown and need to be determined.

To cope with the above-mentioned problems, the signals provided by the accelerometric and magnetic (aiding) sensors are also used [14] as described hereafter. The values provided by the accelerometers correspond to the acceleration the MIMU is subject to, as seen by a non-inertial observer undergoing free-fall. Thus, when the MIMU moves at constant speed or is stationary, the three components of the gravity vector are obtained. Since the components of the same vector in the global frame are known, an orientation (transformation) matrix relating ULF to the global frame can be obtained. It must be noted, however, that this matrix is not unique since rotations about the gravity vector cannot be detected. However, if an axis of the global frame is chosen to match the direction of the gravity vector (GGF), a unique solution for the two Euler angles about the other two axes (often referred to as pitch and roll, or, jointly, as attitude or inclination), can be determined. To obtain information about the third Euler angle, *i.e.*, the orientation of the ULF in the horizontal plane (often referred to as yaw angle or heading), the representation of at least another non-vertical vector is needed in the same GGF. To this purpose, magnetic sensors are used.

The direction of the Earth magnetic field vector varies according to latitude, being aligned with gravity at the poles and perpendicular to it at the equator. For most locations on Earth, both the vertical and horizontal components of the magnetic field are not negligible, although only the latter is needed to provide the complementary information to the accelerometer for heading estimation. Therefore, the attitude estimated from the accelerometer measurements is used to calculate the horizontal components of the magnetic field vector, thus obtaining the heading of the ULF with respect to GGF [15]. As a result, the heading accuracy is necessarily affected by the attitude accuracy.

For practical reasons, both the gravitational and magnetic field vectors are assumed uniform and constant within the measurement volume. While this assumption is easily met by the gravitational field, this is not the case for the magnetic field vector, the magnitude and direction of which may vary due to the presence of ferromagnetic objects or electrical appliances, especially indoors [16]. This is why the estimation of the heading angle is often regarded as more critical than the attitude [17,18]. Additionally, the distortions of the local magnetic field cannot be easily modeled or mapped, leading to systematic errors in the identification of the true North. However, for short distance tracking, as occurring in human movement analysis, these errors are without consequence as the interest lies in the variations in heading with respect to a reference orientation rather than to the true North.

In the light of the previous considerations, the information provided by the three sets of sensors can be combined within a sensor fusion framework. Two main sensor fusion approaches have been proposed in the literature. The first is stochastic filtering, often implemented in the form of an Extended Kalman Filter. Given a model of the time evolution of the state of the system under analysis (the MIMU orientation) and of its noisy observations (the MIMU output signals), Kalman-based methods provide an estimate of that state [19]. The second approach is complementary filtering, which fuses multiple noisy measurements that have complementary spectral characteristics. For each measurement, the complementary filtering exploits only the part of the signal frequency spectrum that contains useful information [20]. In this case, the characteristics of the noise present in the process are not required to be modeled and incorporated in the algorithm.

As highlighted by the extensive literature dealing with the development of the two categories of algorithms [20–30] and their relevant applications in human movement analysis [31–37], the main differences between them consist in: how gyroscope bias drift, inertial acceleration, and magnetic disturbances are modeled/accounted for; how the orientation is described (rotation matrix, unit quaternions, Euler angles); how magnetic sensor data are employed in the estimation of the heading and attitude angles, and, for the stochastic filtering approach, which parameters are included in the model of the state of the system [14].

The problem of the validity and reliability of these methods has been also dealt with in the literature. A large portion of the studies was based on the analysis of the movement of mechanisms operated by a motor (gimbal joint [38], gimbal table [12], robotic arm [20]) or manually (pendulum [39,40], table [27–29,41], tripod [42]). Only few works analyzed movements of human body segments (during sit-to-stand [43], treadmill walking [34,36], eating and morning routine tasks [31,44]), observed for time durations that varied from a few seconds, for the sit-to-stand, to a maximum of 80 s, for the walking task, and of 90 s, for the manual tasks. Movements exhibited small heading variations and were performed in small measurement volumes, and therefore did not allow to test the performance of the algorithms in some of the most challenging conditions.

Despite the reported literature, there is still considerable confusion regarding the actual level of accuracy that can be obtained when estimating the 3D orientation from MIMU measurements in the different possible human movement scenarios. The main open issues are the following: (1) To what extent and in which circumstances the sensor fusion approach is more effective than the numerical integration approach? (2) Does the sensor fusion effectiveness depend on the specific algorithm selected? (3) Given an orientation estimation method, to what extent the overall measurement conditions (e.g., the tracking time duration, the measurement volume, the amplitude of the MIMU inertial acceleration, the presence of large angular movements, and the planarity of the movement) influence its performance?

The purpose of the present work is to answer the above-listed questions for applications in human movement analysis. To this aim, two sensor fusion methods were selected, representative of the stochastic and complementary filtering approaches, and their accuracy was assessed and compared with that of the numerical integration approach. Reference concurrent measurements of orientation were obtained using a stereophotogrammetric system. The three methods were analyzed in two different motor scenarios, the general characteristics of which would be representative of the majority of every-day life movements performed by able-bodied individuals of any age or health status. The signals of a MIMU located on the forearm and generated during daily manual tasks and those of a MIMU located on the pelvis during walking along a curved path were recorded and analyzed, thus providing signals of distinct characteristics to which orientation estimators are most sensitive. As a result, a list of practical recommendations about critical aspects to be taken into consideration to improve MIMU-based 3D orientation accuracy is finally provided.

## 2. Materials and Methods

### 2.1. Subjects and Experimental Set Up

Six subjects (three male and three female, age = 28.6 ± 5.1 years) participated in the study. The research methodology described hereafter was approved by the university institutional review board.

Daily manual tasks were acquired, characterized by a time duration of 60 s, a limited measurement volume (0.8 × 0.8 × 0.8 m^3^), the presence of temporal intervals during which the MIMU was stationary, and rotations occurring about the three MIMU axes exhibiting no prevalence of one degree of freedom with respect to the others. While seated in front of a table with a shelf below it, participants mimicked the following sequence of daily-life activities (*manual routine*): drinking a glass of water (5 s), writing with a pencil (5 s), writing using a keyboard (5 s), brushing teeth (10 s), brushing hair (10 s), reaching towards a small magnet placed on the table (5 s), and moving an object from the table to the lower shelf and back to the table (8 s). During each *manual routine* trial, a static pause of a couple of seconds was included between each of the activities described above.

The second task was level walking (*locomotion*), characterized by a time duration of 180 s, a large measurement volume (4 × 2 × 0.1 m^3^) and the absence of static phases. Each participant was asked to walk at his/her self-selected speed along a “figure of eight” pathway. This pathway, which was determined by two cones located three meters apart, was devised to introduce large rotations in the horizontal plane and to reproduce both left and right turnings. At the beginning of each *manual routine* and *locomotion* trial, participants maintained a static posture for five seconds.

**Figure 1.**
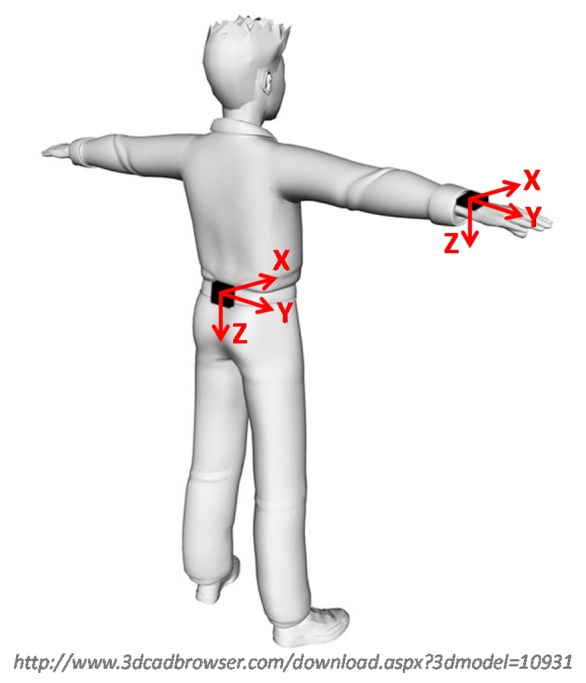
MIMU location and ULF orientation during the static postures (corresponding to the selected GGF): *X* axis, antero-posterior and positive forward; *Y* axis, medio-lateral and positive to the right; *Z* axis, vertically aligned with the direction of the gravitational field vector and positive downwards.

Before the trials, a MIMU (Opal, APDM Inc., Portland, Oregon, USA) containing 3D gyroscopes, accelerometers and magnetic sensors (± 6 *g* with *g* = 9.81 m/s, ± 1500 °/s and ± 600 μT of full-range scale, respectively) was secured using an elastic belt to the participants' lower back (L3–L4) for the *locomotion* trial and to the forearm for the *manual routine* trial (Figure 1). Angular velocity, acceleration and local magnetic field vector data were collected at 128 samples/s.

To validate MIMU-based orientation estimates, four retro-reflective markers were rigidly attached to the unit case and marker trajectories were tracked by a nine-camera stereophotogrammetric system (Vicon MX3, Oxford, UK) at 100 sample/s. MIMU and stereophotogrammetric data streams were synchronized using a square wave signal simultaneously detected by both systems. All data processing was performed with customized functions using the Matlab^®^ software (The MathWorks Inc., Natick, MA, USA).

### 2.2. Stereophotogrammetric and MIMU Data Pre-Processing

Marker trajectories and MIMU measurements were resampled at the same frequency, set at 200 samples/s, using cubic spline interpolation. To remove random noise, marker trajectories were low-pass filtered using a 2nd-order zero-lag Butterworth filter. The cut-off frequency was determined by performing a residual analysis [45] on each trial of each subject. The values obtained were similar among different motor tasks and among different subjects (standard deviation less than 0.3 Hz). Thus, the cut-off frequency value was conservatively set to 6 Hz for all trials.

A marker-cluster local frame (MLF) was defined using the markers attached on the MIMU to obtain its reference orientation with respect to the stereophotogrammetric global reference frame. The time invariant orientation of ULF relative to MLF was estimated following the methodology proposed by Chardonnens *et al.* [46]. The two local frames were thus aligned, by eliminating this time invariant orientation, and rotated so as to have one axis aligned with the vertical line during the static postures, at the beginning of each trial. The 3D orientation of ULF and MLF was then expressed with respect to the same GGF, defined to match the ULF during the static postures (Figure 1). In this way, the changes in orientation of the MIMU were assessed with respect to its own initial orientation. Detailed information about the alignment and rotation procedures, as well as about the definition of the local and global frames, is reported in Appendix A.

Particular attention was paid to the correction of the static bias of the gyroscope signals. Once integrated, in fact, this bias leads to a drift error that grows linearly with time [47]. The bias was calculated as the mean of the gyroscope measurements during a one-minute static acquisition performed by placing the MIMU on a table in the middle of the experimental session, and it was then subtracted from the whole angular velocity time series. Moreover, the calibration of the accelerometers and magnetic sensors was checked at the beginning of the experimental session by performing the *ad hoc* data collection described hereafter. For what concerns the accelerometers, three static trials were acquired for the MIMU, in which each ULF axis was consecutively aligned with the direction of the gravitational field vector for one minute. A plumb line was used to verify the correct alignment of each axis with the vertical line. The average of the signal measured along each axis was computed and its value compared to its expected value (*i.e.*, the gravitational field vector magnitude for the vertical component, and zero, for the two components lying in the horizontal plane). As the maximal difference between the measured and expected accelerations was 0.02 m/s^2^, the accelerometers were considered as properly calibrated. For what concerns the magnetic sensors, the calibration procedure proposed by Gebre-Egziabher *et al.* [48] was followed. The MIMU was freely rotated about its three local axes, and the biases and sensitivities of the magnetic sensors were estimated. These parameters were then used to calibrate the sensor measurements during each trial.

### 2.3. MIMU-Based Orientation Algorithms

The 3D orientation of ULF with respect to GGF was estimated using three different methods. As reported in the introduction section, there are a huge number of MIMU-based orientation estimation algorithms in the literature. However, as most of them fall in the domain of stochastic or complementary filtering approaches, two recent algorithms, representing each approach, were considered and their performances were compared to that of the numerical integration approach.

For all methods, a quaternion parameterization was adopted to describe the MIMU orientation in space with respect to GGF. The superiority of this parameterization with respect to the orientation matrices or the Euler angles representations is widely documented, both in terms of lower computational load [49] and of reduced errors associated with the numerical integration [50]. Since GGF was defined to be aligned with ULF during the static postures at the beginning of each trial, the initial orientation of the MIMU with respect to its own global frame was null. The initial condition for all methods was thus set to the null unit quaternion (***q̄***=[0 0 0 1]*^T^*). It must be specified that all three methods provide the orientation of GGF with respect to ULF and therefore the quaternion was finally transposed to obtain *^GGF^**q̄**_ULF_*.

#### 2.3.1. Numerical Time-Integration Method (INT)

The INT method was based on the numerical time integration of the differential kinematic equation describing the relationship between the quaternion derivative and the angular velocity components measured by the gyroscope (*ω_X_*,*ω_Y_*,*ω_Z_*) [14,51]:

(1) $$\frac{d}{dt}\bar{q}=\frac{1}{2}{\left[\omega_{\text{ULF}}^{T}0\right]}^{T}⊗\bar{q}=\frac{1}{2}\left[\begin{array}{cc} -\left[\omega_{\text{ULF}}\times \right] & \omega_{\text{ULF}}\\ -\omega_{\text{ULF}}^{T} & 0 \end{array}\right]\bar{q}=\Omega \left(\omega_{\text{ULF}}\right)\bar{q}$$

where $\bar{q}={\left[\begin{array}{cc} q^{T} & q_{4} \end{array}\right]}^{T}$ is the quaternion representation of the rotation from the global to the local frame and is composed of a vector part ***q*** and a scalar part *q*_4_, whereas ⨂ is the quaternion product operator; *ω_ULF_* is the angular velocity vector measured by ULF, [*ω_ULF_* ×] is the skew-symmetric matrix of *ω_ULF_*, and **Ω**(*ω_ULF_*) is the compact notation for the resulting 4 × 4 skew-symmetric matrix. It is worth noting that Equation (1) does not involve computationally expensive non-linear trigonometric functions and it is not affected by the presence of singularity points, in contrast to orientation parameterizations such as the Euler angles. The discrete-time equivalent of Equation (1) [14] is analytically integrated by assuming that the angular velocity signals are constant within each interval of time between two subsequent samples.

#### 2.3.2. Stochastic Filtering Method (SF)

**Figure 2.**
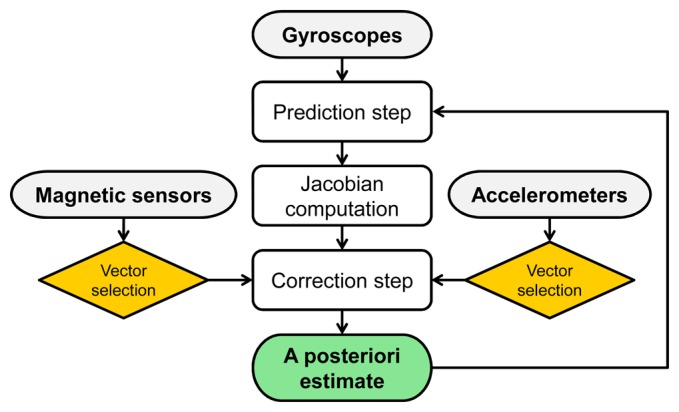
Framework of the SF method.

The quaternion-based Extended Kalman Filter presented by Sabatini [14] was selected as representative of the stochastic filtering approach. On the one hand, state propagation (*i.e.*, quaternion propagation) is performed through Equation (1), where **Ω**(*ω*) is obtained from gyroscope measurements. On the other hand, accelerometers and magnetic sensors are used to prevent the drift resulting from the numerical integration of Equation (1) and are both involved in the estimation of the heading angle (Figure 2). Before performing the Kalman update step, measurements are verified in terms of expected fields magnitudes and directions. Online magnetic sensor bias estimation is implemented to cope with undesired variations of the local reference magnetic field.

In general, all stochastic filtering approaches take the sensor measurements reliability into account. Data confidence is typically quantified in terms of standard deviation of the measurement noise that is required to be specified in the method. These standard deviations are then used to determine the weight assigned to each input measurement when estimating the state of the system. In the present work, eight parameters were considered and, based on the different characteristics of the tested motor tasks and on the results of a trial-and-error procedure, two different sets of parameters were defined for the *manual routine* and *locomotion* tasks. Their values are reported in Table 1.

#### 2.3.3. Complementary Filtering Method (CF)

**Figure 3.**
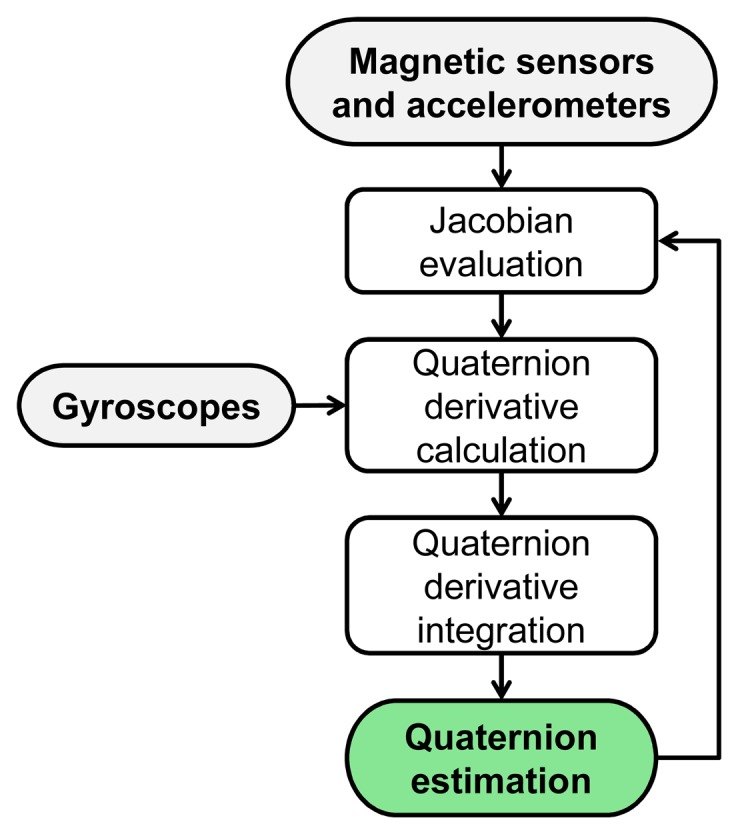
Framework of the CF method.

The non-linear observer proposed by Madgwick *et al.* [27] is a recent example of the complementary filtering technique and was selected as representative of this kind of approach (Figure 3).

Basically, Equation (1) is extended with a term derived from the accelerometer and magnetic sensor measurements, which is computed using a single-iteration minimization algorithm. Ferromagnetic disturbances are dealt with by defining a time varying representation of the local magnetic field, with a null component along the Y-axis. This procedure allows the magnetic sensors to provide an estimate of the heading angle while not affecting the attitude. It is worth to underline that, contrary to SF, no information about the noise characterizing the process is taken into account in the CF method, and no selective thresholds are set on the use of the accelerometer and magnetic sensor measurements.

The only tuning parameter required for the CF method is *β*, which represents the gyroscope measurement errors and can be estimated as follows:

(1) $$\beta =\sqrt{\frac{3}{4}}{\tilde{\omega }}_{\text{max}}$$

where ***ω̃****_max_* is the maximum gyroscope error (equal to three times the gyroscope noise standard deviation). For the gyroscopes of the MIMU used in the present study, the resulting *β* value was about 1.15 °/s. Similarly to what performed for the SF method, *β* was modified according to the results of a trial-and-error procedure, and a final value of 0.1 rad/s (corresponding to 5.73 °/s) was selected for both the *manual routine* and the *locomotion* tasks. This value is in accordance with the default value set by Madgwick *et al.* in the open-source Matlab® implementation of the method (available at http://www.x-io.co.uk/open-source-imu-and-ahrs-algorithms/, last accessed 18 September 2014).

### 2.4. Orientation Accuracy Assessment

**Figure A1.**
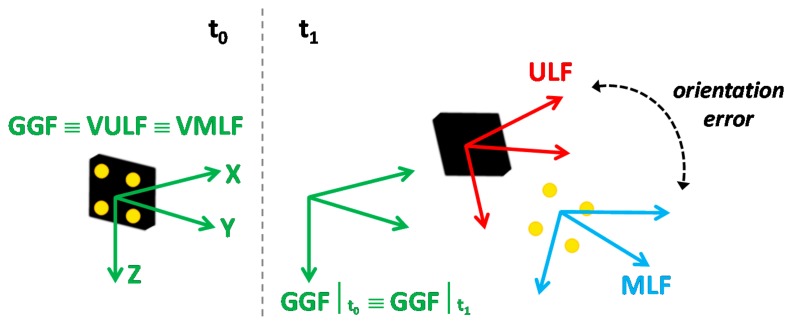
MIMU, marker and global frames are depicted during the static postures at the beginning of each trial (t_0_) and in a generic time instant during the trial (t_1_). The relative orientation of ULF with respect to MLF which corresponds to the orientation error is also indicated at t_1_. For clarity reasons, the distance between the origins of MLF and ULF has been emphasized.

For the *manual routine* and the *locomotion* tasks, the accuracy of INT, SF and CF in estimating the orientation of the MIMU was evaluated by computing the error quaternion expressing the orientation of ULF with respect to MLF for each method: Δ***q̄*** = *^MLF^**q̄**_ULF_* [14,17] (see Figure A1 in Appendix A). The orientation error is obtained from the scalar component of Δ***q̄*** according to the equation: Δθ=2cos^–1^(Δ*q*_4_). The accuracy of each method was then expressed in terms of the Root Mean Square (RMS) value of the orientation error. It should be noted that the stereophotogrammetric errors [52] propagate to the angles of interest in this study causing a maximal inaccuracy of 0.5°.

Because of the different contribution provided by the accelerometer and the magnetic sensor measurements in the estimation of the heading and attitude errors [17,31,53], the quaternion error was decoupled into two components: the heading error – *RMS_head_* – and the attitude error – *RMS_att_*. A detailed description of the procedures to decouple the orientation error into these two components can be found in Appendix B.

### 2.5. Statistical Analysis

For each motor task and each method, the normal distribution of *RMS_head_* and *RMS_att_* was verified using the Shapiro-Wilk test of normality. Both error parameters relative to the *manual routine* were not normally distributed, whereas their distribution for the *locomotion* task was found to be normal. According to these results, non-parametric and parametric tests, respectively, were selected in order to answer the following questions:

- Is there any difference in the level of accuracy between the sensor fusion methods and the numerical integration approach? And, is there any difference between the accuracy of the SF and CF methods? To investigate the effect of the “method” factor, the Friedman test was performed separately on *RMS_head_* and *RMS_att_* for the *manual routine*. When a significant “method” effect was found, pairwise comparisons were performed using the Mann-Whitney U test with the Sidak correction, after having verified the assumption of equality of variance between each pair of data. Similarly, for the *locomotion* task, a repeated measure one-way Analysis of Variance (ANOVA) was performed. The sphericity assumption was tested using the Mauchly test and the Greenhouse-Geisser correction was applied if this assumption was violated. If a significant *p*-value was reported for the “method” effect, the Sidak *post hoc* pairwise comparisons were performed using the relevant correction. The partial Eta squared measures were also computed, for each dependent variable, to give the proportion of variance accounted for by the “method” factor.
- Is there any difference in the orientation accuracy between the *manual routine* and the *locomotion* tasks? For each tested method, an independent Mann-Whitney U test was performed separately on the *RMS_head_* and *RMS_att_* obtained in the *manual routine* and *locomotion* tasks, in order to investigate whether significant differences exist between the levels of accuracy of the two scenarios.

The alpha level of significance was set to 0.05 for all statistical tests. The statistical analysis was performed using IBM SPSS Statistics software package (IMB SPSS Statistics 21, SPSS IBM, New York, NY, USA).

## 3. Results

### 3.1. Method Comparison

**Figure 4.**
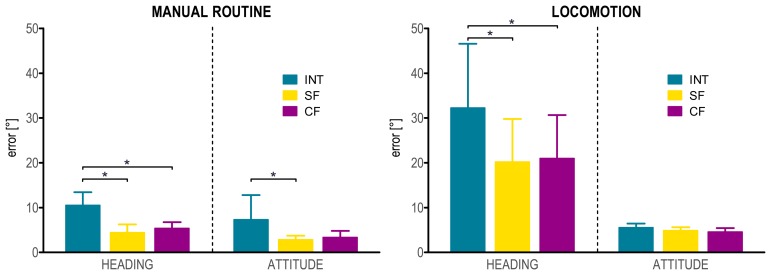
Heading and attitude errors (mean and one standard deviation) for the *manual routine* (on the left) and the *locomotion* (on the right) tasks. Significant differences among the INT, SF and CF methods are indicated with an asterisk.

For the *manual routine*, a significant effect of the “method” factor was observed for the heading error *RMS_head_* ( $\chi_{6}^{2}=12$, *p* < 0.001). Mann-Whitney's U test revealed significant differences between INT and both SF and CF (*U_6_* = 0, *p* < 0.005, for both methods). Conversely, no significant difference was obtained between SF and CF. When the attitude error *RMS_att_* was considered, a significant “method” effect was found ( $\chi_{6}^{2}=7$, *p* < 0.05). Pairwise comparisons showed significant differences only between INT and SF (*U_6_* = 4, *p* < 0.05), while no difference was found neither between INT and CF nor between SF and CF (Figure 4).

For the *locomotion* task, the ANOVA analysis showed a significant effect of the “method” factor for *RMS_head_* (*F_2,10_* = 21.678, *p* < 0.001). The partial Eta squared was 0.81, indicating that the “method” factor by itself accounted for 81% of the overall variance in the dependent variable *RMS_head_*. *Post hoc* tests revealed significant differences between INT and both SF and CF (*p* < 0.05 for both methods). For the attitude error *RMS_att_*, a significant “method” effect was also found (*F_2,10_* = 5.255, *p* < 0.05), with a partial Eta squared of 0.51. However, according to the *post hoc* comparisons, no significant differences were observed between each pair of methods (Figure 4).

**Figure 5.**
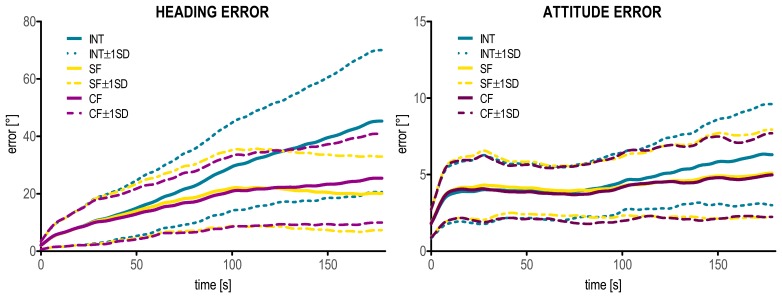
Heading and attitude errors (Δθ) of the INT, SF and CF methods plotted as a function of time for the *locomotion* task. The mean ± one standard deviation (SD) curves over the six participants are reported. Note the different scale of the axes of the ordinate in the two graphs.

In Figure 5 the behavior of the heading and attitude errors as a function of time is depicted, during the three minutes *locomotion* task and for the three methods.

### 3.2. Task Comparison

When comparing the performance of each method between the *manual routine* and the *locomotion* tasks, significant differences were found on *RMS_head_* for INT, SF and CF. In particular, for all methods, heading error values during the *manual routine* task proved to be significantly smaller than those obtained during the *locomotion* task (INT: *U_6_* = 1, *p* < 0.005; SF: *U_6_* = 1, *p* < 0.005; CF: *U_6_* = 2, *p* < 0.005). When considering *RMS_att_*, only SF performed significantly better during the *manual routine* trials with respect to the *locomotion* task (*U_6_* = 1, *p* < 0.005). Conversely, no difference was reported between the *manual routine* and the *locomotion* scenarios for INT and CF.

## 4. Discussion

In the present study, two sensor fusion methods for the estimation of 3D orientation using MIMUs were selected as representative of the stochastic and complementary filtering approaches, and their performance was compared with that of the numerical integration method. The three algorithms were analyzed during manual and locomotion tasks, and their level of accuracy was assessed, in terms of heading and attitude errors, with respect to reference orientations obtained through stereophotogrammetry.

Average heading errors during the *manual routine* task were lower than 5.5° for the two sensor fusion approaches and lower than 10.5° for INT. Similar results were obtained for the attitude errors (*RMS_att_* < 3.5° and *RMS_att_* < 7.3°, for the sensor fusion methods and INT, respectively). These results are in agreement with the findings of two previous studies published by Luinge *et al.* [31,44], where the 3D orientation of an inertial sensor located on the forearm was estimated during both eating and morning routine tasks using an *ad hoc* developed Kalman filter. However, no magnetic sensors were included in both studies, therefore further comparison with the present work would not be appropriate.

When considering the *locomotion* task, average heading errors increased up to 21° for the sensor fusion methods and to 32° for the INT method, while average attitude inaccuracies remained lower than 5.5° in all cases. Also in this case, a thorough comparison with previous studies can be hardly performed, due to the variety of experimental protocols and methodologies used to assess the accuracy of the analyzed methods or devices. In one study [32], participants were asked to walk in a straight line for 10 m and trunk, thigh and shank inclination in the sagittal plane was estimated using a non-linear filter. Trial duration was approximately 4 s and RMS differences of 1.5° and 3.0° between reference and estimated angles were found for the trunk and the shank segments, respectively. In other studies, the orientation of a lower trunk mounted inertial sensor during treadmill walking was obtained by using an *ad hoc* developed Kalman filter [34] or a Weighted Fourier Linear Combiner adaptive filter [36]. Participants walked at natural, slow and fast speeds and trial duration was about 40 s [34] and 80 s [36]. RMS differences between reference and estimated attitude angles were lower than 1° in both studies. Only one study [36] estimated the heading angle, in which RMS values between reference and estimated angles were reported to be lower than 1.5°. Related to the range of motion considered in these studies, the low absolute values of these errors actually account for about 10% of the angular displacement, as no change of direction was performed. It is plausible that the larger errors obtained in the present study can be attributed to the longer time durations and to the larger measurement volumes involved, as well as to the large angular movements occurring on the horizontal plane (“figure of eight” pathway with rotations of about 260°).

### 4.1. Method Comparison

#### 4.1.1. Heading Angle

When comparing the performance of the three methods in the estimation of the heading angle, the SF and CF methods performed significantly better than the INT method, during both the *manual routine* and *locomotion* tasks. This result indicates that the sensor fusion approach is successful in limiting drift errors of the numerical integration approach when the heading angle is concerned, even when long time durations, large measurement volumes, no static or quasi-static phases, and changes of direction are involved. In particular, the time-error curves (Figure 5) show that the contribution of the sensor fusion methods starts to be considerable approximately after the first 20 s. Under this threshold, the performances of the three methods are essentially indistinguishable. Based on this result, it is clear that to analyze the benefit of sensor fusion methods in the accuracy of MIMU-based orientation estimation, motor tasks characterized by short time durations (few seconds) are not recommendable. Nevertheless, it must be noted that the motor tasks selected in this study do not involve impacts (e.g., as in jumping or clapping), in which case the benefit of a sensor fusion approach could be appreciable also in motions of short duration.

It is also interesting to note that, during the *manual routine*, the subjects were asked to move their forearm towards a small magnet placed on the table. No detrimental effects were observed in the performances of SF and CF when the MIMU approached the magnet, indicating that both algorithms are effective in identifying and compensating sudden and relatively high ferromagnetic disturbances (B = 200 μT, equivalent to about five times the magnitude of the measured magnetic field vector).

#### 4.1.2. Attitude Angle

Different results were obtained for the attitude angle, for which the contribution of the sensor fusion methods was evident only during the *manual routine* task. In particular, both SF and CF methods showed a higher level of accuracy with respect to INT (about 60% and 50% of error reduction for the two methods, respectively). However, this difference was statistically significant only for SF. This is probably due to the different assumptions that SF and CF make with respect to the use of the magnetic sensor data: SF uses these data to estimate both the heading and attitude angles, whereas CF relies on magnetic measurements only for the heading estimates. It is plausible that, as far as small measurement volumes are concerned, the magnetic field is reasonably uniform and constant, thus making it convenient to rely on magnetic sensor data to estimate both the heading and the attitude angles.

The results obtained for the attitude error during the *locomotion* task were somehow unexpected. No statistical difference between INT and the sensor fusion methods were found, probably because INT performs remarkably well providing an average *RMS_att_* smaller than 5.5° and displaying a reduced drift error even after three minutes of numerical integration (Figure 5). Further investigation is needed in order to better understand which factors are actually involved in determining the amount of drift error associated with the integration of the gyroscope signals. However, the present results suggest that this drift depends not only on the time duration and on the standard deviation of the noise underlying the gyroscope signals [13], but also on the amplitude of the angular velocity itself. It can be speculated, therefore, that the reduced drift error associated with the attitude angle estimation are related to the reduced angular velocity values measured about the *X* and *Y*-axes during walking. In fact, during the *locomotion* trials, there is an uneven distribution of rotations about the three MIMU axes: in particular, the angular velocity about the vertical axis (more involved in heading estimation) was much higher (about 3.6 times) with respect to the other two axes (involved in the attitude assessment). Similar considerations can be drawn for the sensor fusion methods, as they both rely on the numerical integration of the gyroscope signals.

#### 4.1.3. Sensor Fusion Methods Comparison

For both motor tasks and both error components, no statistical difference between SF and CF performance was found, indicating that the two methods can be considered equally effective in limiting drift errors of the numerical integration approach, within the scenarios analyzed in the present work. It cannot be excluded that in other contexts the two methods would perform differently, for instance when impacts or time durations largely exceeding three minutes are involved. The two methods however present different strengths: on the one hand, the complementary filtering approach has the advantage of requiring the tuning of only one parameter and entails a reduced computational load; on the other hand, Kalman-based approaches allow for considerable freedom in customizing the models used to describe both the time evolution of the system state and the observations, including the noise characteristics of each variable. This freedom can be exploited to conceive different variants of Kalman filters, with several opportunities available to fine-tune the filter structure. It was, however, far beyond the aims of the present study to provide a conclusive answer with respect to which contextual factors and implementation details determine the performance of each tested method. In this respect, a Monte Carlo simulation approach would be very well suited.

### 4.2. Task comparison

#### 4.2.1. Heading Angle

As expected, heading errors obtained during the *manual routine* were significantly lower than those obtained in the *locomotion* task, for all the three methods. Different factors, which differentiate the *locomotion* task with respect to the *manual routine*, are assumed to explain this result: first, the longer time duration, which entails larger drift errors associated with the numerical integration of the gyroscope signals; second, the larger measurement volume, which affects the reliability of the magnetic sensor measurements due to the magnetic field vector variation, which may be too small and smooth to be identified as a disturbance by the sensor fusion methods; third, the absence of temporal intervals during which the MIMU was stationary, which influences the possibility to use the accelerometer as an aiding sensor to reduce the above mentioned drift errors; fourth, the large angular movements occurring in the horizontal plane and the uneven distribution of rotations about the three MIMU axes, which seems to be implicated in a reduction of the performance of both the INT and the sensor fusion methods [12].

#### 4.2.2. Attitude Angle

For the attitude errors, only SF performs significantly better during the *manual routine* with respect to the *locomotion* task. In this respect, it is interesting to note that, although for the *manual routine* the heading and the attitude errors present similar values, a large difference exists between the two error components for the *locomotion* task. This can be explained by two factors: (i) the similar angular velocity amplitude measured about the three MIMU axes in the *manual routine*, against the higher angular velocity measured about the vertical axis with respect to the other two axes, during the *locomotion* task; (ii) the critical estimation of the heading with respect to the attitude angle when dealing with the sensor fusion approach [15,18], especially in challenging conditions like those characterizing the *locomotion* task. Furthermore, it is worth noting that both *RMS_head_* and *RMS_att_* should be compared to the angular range of motion of the heading and attitude angles, respectively. As previously mentioned, in the *manual routine*, the amount of rotations was similar about the three axes (about 120°). Conversely, during *locomotion*, the angular displacement about the vertical axis was about 260°, whereas that about the other two axes was less than 10°. Although the average value of the attitude errors is about 5°, this error is in the same order of magnitude of the total range of movement occurring on the frontal and sagittal planes. In other words, although no statistical differences were found between the *manual routine* and *locomotion* attitude errors, care should be paid to the relative impact that these errors have on each task. Similar considerations can be drawn for the heading errors.

## 5. Guidelines

**Figure 6.**
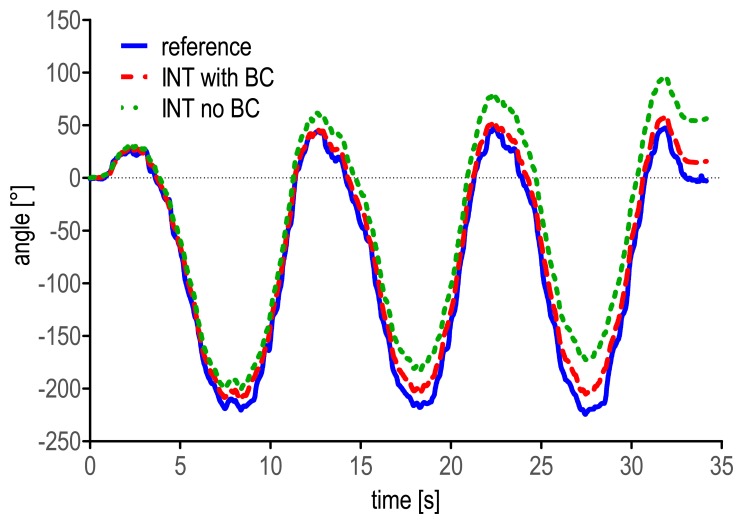
Heading angle as obtained during three complete “figures of eight” of one randomly selected *locomotion* trial. Angles obtained by the stereophotogrammetric system (solid line), and by numerical integration with (dashed line) and without (dotted line) bias correction (BC) are depicted.

**Figure 7.**
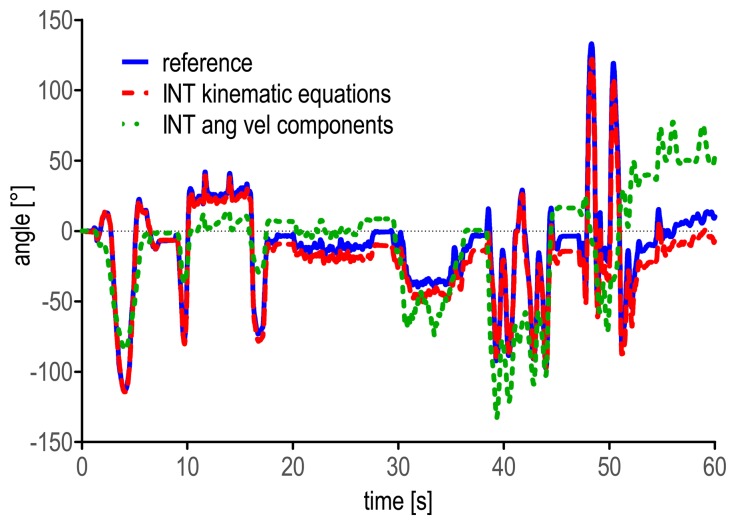
Heading angles as obtained by the stereophotogrammetric system (solid line) and by integration of the kinematic equations (dashed line) are depicted together with the values obtained by integration of the Z component of the angular velocity (dotted line) measured by the MIMU on the forearm, during one randomly selected *manual routine* trial.

As resulting from the previous considerations, several critical aspects are involved in the accurate estimation of 3D orientation from MIMU data. A list of practical recommendations has thus been formulated with the aim of limiting those error sources that can be taken into account *a priori*. It is strongly advised to pay attention to the following aspects:

- *Correct the gyroscope static bias*. Special attention should be paid to the correction of the initial bias of the gyroscopes. It is recommended first to switch on the MIMU at least 20 min before the beginning of the acquisitions to ensure that the device will have reached its working temperature when the experimental session starts, thus avoiding wide variations of the static bias due to heating effects [13]. Second, at least one static acquisition should be performed to compute the bias of the gyroscopes: if, after the MIMU positioning on the participants, a static phase can be identified in which the unit is reasonably still, such data can be employed for bias correction; otherwise the MIMU can be placed on a stable surface (e.g., on a table). The optimal duration of the static phase may depend on the electronics of the employed devices and, clearly, on the sampling frequency. However, to the authors' experience, five seconds at a sample rate of 100 samples/s can be considered as a sufficient duration. As an example of the influence of the bias correction procedure on the outcome accuracy, Figure 6 displays the heading angle of the MIMU on the lower trunk with and without the static bias correction, during three complete “figures of eight” of one selected trial of the *locomotion* task.
- *Check if the accelerometers and the magnetic sensors are properly calibrated*. Careful attention should be paid to the calibration of the accelerometers and magnetic sensors. Different procedures are available in the literature aiming at verifying and, possibly, calibrating the accelerometers [54–56] and the magnetic sensors [48]. In both cases, *ad hoc* experiments should be performed to estimate the biases and sensitivities of the sensors under analysis. This information can then be used to correct the measurements obtained during the experimental sessions. Moreover, ferromagnetic disturbances can be reduced by following the safe procedures described by De Vries *et al.* [57] and Bachmann *et al.* [16].
- *Integrate the kinematic equations, not just the components of the angular velocity signals*. The kinematic differential equations that describe the relationship between the time derivatives of the selected orientation parameters and the angular velocity components (e.g., Equation (1) for quaternion representation) describe not only the changes in orientation of the local reference frame with respect to the previous one in each instant of time, but also the orientation between the local and the global reference frames in each instant of time. The latter information is missing when the numerical integration is performed on the components of the angular velocity signal separately [51]. Additionally, the angular displacements obtained in such a way do not represent Euler angles. When out-of-plane movements or large rotations about at least one of the three MIMU local axes are addressed, the piece of information neglected by the individual component integration can be crucial. Only when small rotations are involved and, thus, negligible changes in orientation between the local and the global reference frames occur, the results provided by the integration of the angular velocity components are comparable to those obtained by integrating the kinematic equations (this is the case, for example, of straight treadmill or over-ground walking using a MIMU placed on the lower trunk). Nevertheless, the integration of the kinematic equations should be always preferred, as more rigorous. As an example of the different results that can be obtained using the two integration methods, the heading angle obtained by integrating the kinematic equations and the angular displacement obtained by integrating the Z component of the angular velocity signal are reported during one randomly selected *manual routine* trial (Figure 7).
- *Be careful when comparing MIMU-based estimates to validation measures*. Regardless of the instrument used to validate MIMU-based orientation estimates, special attention should be paid to the initial alignment between the local systems of reference of the validation instrument and the MIMU. In case motion capture systems are used, different procedures exist that align the marker-based and the MIMU local frames by means of *ad hoc* acquisitions [46,58]. Furthermore, the validation instrument and the MIMU local systems of reference must be expressed in the same global reference frame. Usually the global frames of the two devices do not match, and therefore, the relative orientation between them is needed to define a common global system of reference.

## 6. Conclusions

In the present paper, a number of critical aspects related to the accurate estimation of 3D orientation from MIMU data in typical human movement analysis scenarios have been discussed and a set of structured guidelines have been provided as a useful outcome and code of practice for the scientific community working in the field.

The sensor fusion approach has proven effective in compensating the limitations associated with the use of the gyroscopes alone, particularly when the measurement conditions do not challenge the main limitations of the aiding sensors (*i.e.*, variations of the local magnetic field vector for the magnetic sensor, and inertial accelerations for the accelerometer). This effectiveness does not depend on the sensor fusion approach selected, at least for the SF and the CF methods tested in the present study.

Among the factors affecting the accuracy of MIMU-based 3D orientation estimation, the following are particularly noteworthy: time duration, measurement volume, and presence/absence of phases during which the MIMU is stationary. In addition, the distribution of rotations, as well as the amplitude of angular velocity about the three MIMU axes, seems to play a relevant role. Their combined effect has been assessed in the present study, however further investigation should be carried out to establish the individual role of the above-mentioned factors in determining the outcome accuracy.

A comprehensive comparative assessment of all published algorithms developed for the estimation of 3D orientation from MIMU measurements is virtually impossible. The same applies to the variety of applications, even when limited to the context of human movement analysis. Nevertheless, the two main families of sensor fusion approaches have been considered and state-of-the-art algorithms have been selected as their representatives. Additionally, the motor tasks employed in the study not only represent the majority of every-day life movements, but also cover a wide range of challenging acquisition conditions to which MIMU-based orientation estimation methods are most sensitive.

The general validity of the results of the present study, however, does not imply that the relevant conclusions can be straightforwardly applied to any context. Impacts, for instance, have not been considered, and their effect on the performance of sensor fusion methods may represent a challenge which requires further investigation. Moreover, the results cannot be directly extended to applications other than movement analysis (e.g., aircraft navigation) as they present features and challenges of a different nature.

## Figures and Tables

**Table 1.** Input parameters for the SF method.

	**Manual Routine**	**Locomotion**
**Process noise statistics**		
Gyro standard deviation [°/s]	2.5	2.5
Gyro bias process noise standard deviation [°/s^2^]	0	0.01
Magnetic variations process noise standard deviation [μT/s]	1	10
Magnetic variations process noise correlation time [s]	1	1
**Measurement noise statistics**		
Accelerometer standard deviation [*g*/10^3^]	10	2.5
Magnetic sensor standard deviation [μT]	3	3
**Threshold for vector selection**		
Acceleration measurements [*g*/10^3^]	40	10
Magnetic sensor measurements [μT]	5	5

## Appendix A

In this appendix, the procedures followed to align the marker (MLF) and the unit local frame (ULF), to rotate them so as to have one axis aligned with gravity, and to define the global reference frame (GGF) with respect to which both MLF and ULF orientations were expressed, are presented.

MIMU and marker-based orientations were represented using quaternions (***q̄***) and orientation matrices (**R**). Under the convention adopted in the present work, *^GGF^***q***_LF_* and *^GGF^***R***_LF_* indicate the relative orientation of the local frame (LF) with respect to GFF and, therefore, allow a coordinate transformation between the two frames.

### A.1. Alignment of the Marker-Cluster and the MIMU Local Frames

The MLF and the ULF were made as parallel as possible to each other by manually aligning the markers with the unit plastic case. However, this does not guarantee that MLF and ULF are perfectly aligned. Moreover, no information is provided by the MIMU manufacturer about the accuracy of the alignment of each single sensor with the unit case. Therefore, the time invariant orientation of ULF relative to MLF (*^GGF^***R**_*ULF*_) was estimated following the methodology proposed by Chardonnens *et al.* [46]. The general concept of this method is to estimate the angles obtained by the numerical integration of each angular velocity component for both MLF and ULF, while performing rotations around three orthogonal axes, and then to use an optimization algorithm to calculate the alignment matrix between the two local frames.

### A.2. Vertical MIMU and Marker-Cluster Local Frames

Both ULF and MLF were rotated so as to have one axis aligned vertically during the static postures at the beginning of each trial. In particular, the resulting vertical systems of references (VULF and VMLF) were oriented as follows: Z axis vertical, aligned with the gravity line and positive downwards; X axis antero-posterior and positive forwards; Y axis medio-lateral and positive to the right (Figure A1). To this aim, the inclination of ULF with respect to gravity was computed during the static postures at the beginning of each trial using accelerometer measurements following the yaw-pitch-roll (*ψ*,*θ*,*φ*) rotation sequence [14]:

(A1) $$\begin{array}{c} \psi_{Z}=0\\ \vartheta_{Y}=-\sin^{-1}\left(\frac{acc_{X}}{g}\right)\\ \varphi_{X}=\tan^{-1}\left(\frac{acc_{Y}}{acc_{Z}}\right) \end{array}$$

where *acc_X_*, *acc_Y_* and *acc_Z_* are the acceleration components as measured by the MIMU and *g* is the norm of the gravitational acceleration. Thus, VULF was defined as ULF rotated by the angles *φ_X_*, *θ_Y_* and *ψ_Z_* The matrix describing the orientation of ULF with respect to VULF (*^VULF^***R***_ULF_*) was then obtained [14]:

(A2) [RVULFULF]=[cϑYcψZcϑYsψZ−sϑYsφXsϑYcψZ−cφXsψZsφXsϑYsψZ+cφXcψZsφXcϑYcφXsϑYcψZ+sφXsψZcφXsϑYsψZ−sφXcψZcφXcϑY]

where *c* and *s* are compact notation for *cos* and *sin*, respectively. The MIMU data (*k*), measured with respect to ULF, were finally expressed with respect to VULF as follows:

(A3) [kVULF(t)]=[RVULFULF]⋅[kULF(t)]

Similarly, assuming that VULF coincided with the vertical marker-cluster local frame (VMLF) while subjects were still at the beginning of each trial, the inclination of MLF with respect to VMLF was estimated during the static posture time lapse:

(A4) [RVMLFMLF]=[RVULFULF]⋅[RMLFULF]T

### A.3. Inertial Global Frames Definition

The vertical marker-cluster local frame (VMLF) and the vertical unit local frame (VULF) were expressed with respect to the same GGF, defined to match both VULF and VMLF during the static postures. In this way, the changes in orientation of VMLF and VULF were assessed with respect to their own initial orientation. To this aim, the time-invariant orientation between the stereophotogrammetric global frames (SGF) and GGF (*^GGF^***R***_SGF_*) was obtained during the static postures:

(A5) [RGGFSGF]=[RGGFMLF]⋅[RSGFMLF]T

where *^GGF^***R**_*MLF*_ was obtained through Equation (A4) as VMLF coincides with GGF during the static postures. Subsequently, the orientation of MLF with respect to GGF during the motion was estimated in each instant in time:

(A6) [RGGFMLF(t)]=[RGGFSGF]⋅[RSGFMLF(t)]

Finally, the orientation of VMLF was obtained with respect to GGF:

(A7) [RGGFVMLF(t)]=[RGGFMLF(t)]⋅[RVMLFMLF]T

## Appendix B

In this appendix, the procedures used to decouple the orientation error Δθ into the heading and attitude components are described.

For each method and each motor task, the error quaternion was first computed as follows:

(B1) Δq¯=q¯VMLFVULF=q¯GGFVMLF−1⊗q¯GGFVULF

where Δ***q̄*** represents the orientation of VULF with respect to VMLF.

Second, Euler angles were extracted from Δ***q̄*** following the yaw-pitch-roll (*ψ*,*θ*,*φ*) rotation sequence, and Δ***q̄****_head_* was set to the quaternion corresponding to the yaw angle error:

(B2) $$\Delta {\bar{q}}_{\text{head}}=[\begin{array}{cccc} 0 & 0 & \sin(\psi /2) & \cos(\psi /2) \end{array}]$$

whereas the contribution of the pitch and roll angles was assembled in Δ***q̄****_att_*, which was calculated as the product of the quaternions representing the pitch and roll angle errors, respectively:

(B3) $$\Delta {\bar{q}}_{\text{att}}=[\begin{array}{cccc} 0 & \sin(\vartheta /2) & 0 & \cos(\vartheta /2) \end{array}]⊗[\begin{array}{cccc} \sin(\varphi /2) & 0 & 0 & \cos(\varphi /2) \end{array}]$$

The orientation errors Δθ*_head_* and Δθ*_att_* were then obtained from the scalar components of Δ***q̄****_head_* and Δ***q̄****_att_*: respectively:

(B4) $$\begin{array}{ccc} {\Delta \theta }_{\text{head}}=2\cos^{-1}(\Delta q_{4\text{head}}) & \text{and} & {\Delta \theta }_{\text{att}}=2\cos^{-1}(\Delta q_{4\text{att}}) \end{array}$$

The accuracy of each method was finally expressed in terms of the Root Mean Square (RMS) value of the orientation errors: *RMS_head_* and *RMS_att_*.

## Abbreviations

MIMU: magnetic and inertial measurement unit
GGF: Global Earth-fixed frame with one axis aligned with gravity
ULF: MIMU local frame
MLF: marker-cluster local frame
INT: numerical time-integration method
SF: stochastic filtering method (Extended Kalman Filter)
CF: complementary filtering method (Non-linear observer)
